# Why people donate their brain to science: a systematic review

**DOI:** 10.1007/s10561-019-09786-3

**Published:** 2019-09-19

**Authors:** Meng-Jiun Penny Lin, Tanisha Jowsey, Maurice A. Curtis

**Affiliations:** 1 School of Curriculum and Pedagogy, Faculty of Education and Social Work, The University of Auckland, Auckland, New Zealand; 2 Faculty of Medical and Health Sciences, Centre for Medical and Health Sciences Education, The University of Auckland, Auckland, New Zealand; 3 Anatomy and Medical Imaging, Faculty of Medical and Health Sciences, The University of Auckland, Auckland, New Zealand

**Keywords:** Brain donation, Donor, Attitudes, Motivations, Barriers, Review, Meta-ethnography, Framework for Assessing Qualitative Evaluations (FAQE)

## Abstract

The acquisition of brain tissue for research purposes is an important endeavour in research on ageing, pathological diagnosis, and the advancement of treatment of neurological or neurodegenerative diseases. While some tissue samples can be obtained from a living patient, the procurement of a whole brain requires the donation from people after their death. In order to promote positive attitudes towards brain donation, it is essential to understand why people do or do not donate their brain to medical research. In 2018 we undertook a systematic review of the international literature concerning people’s attitudes, motivations, and feelings about brain donation. Five electronic databases were searched: Scopus, PsycINFO, Embase, Medline, and Google Scholar. Search terms included: (“brain donor*” OR “brain donation” OR “brain banking” OR “banking on brain”) AND (attitude* OR motivation* OR decision*”) AND (LIMIT-TO “human”) AND (LIMIT-TO (LANGUAGE, “English”)). Articles were analysed using the Framework for Assessing Qualitative Evaluations and a meta-ethnographic approach. Fourteen articles were included for review. The findings suggest four universal factors informing a person’s decision to donate their brain: (1) contextual knowledge, (2) conceptual understandings, (3) family/friends matter, and (4) personal experience, time and process. The findings also indicate that the way healthcare professionals present themselves can influence people’s feelings and attitudes towards brain donation. Healthcare and research professionals who are involved in brain donation processes must be mindful of the complex and multiple factors that influence donation outcomes. Effective and sensitive communication with potential donors and their family/friends is paramount.

## Introduction

Advances in medical science often stem from research conducted on human tissue and organs. There is an ever-present tension between the importance of generating sufficient tissue, samples or specimens for “societal and ethically crucial goods and the rights of individuals or their families to control the use of such material” (Price 2010, p. 3). Notwithstanding, donated organs and tissue offer incalculable benefits to humankind for therapeutic or research purposes (Azizi et al. 2006; Eatough et al. 2012; Harris et al. 2013; Price 2010). Without reservation, brain donors are important. Their donated brains can provide vital clues about neurodegenerative diseases, and new studies can potentially advance treatment. Typically, brain banks depend heavily on donation awareness among potential donors and relatives (Eatough et al. 2012). Information about the importance of brain donation is usually provided through related organisations’ newsletters, speeches by researchers, and community outreach programmes (Eatough et al. 2012; France et al. 2017).

While ample studies reveal the motivations of transplant donors, less is known about the factors motivating people to donate organs for research purposes (Kuhta et al. 2011). People with neurological disorders or mental illnesses are often motivated to donate their brain after death (Azizi et al. 2006; Boise et al. 2017; Boyes and Ward 2003; Harris et al. 2013; Lambe et al. 2011). In contrast, there has been a critical shortage of neurologically healthy brains donated, and these brains are important for comparative work and understanding normal brain processes (Schmitt et al. 2001). Globally, public awareness and knowledge about brain donation is low. Most people are more familiar with the concept of organ donation for transplantation than of brain donation. Researchers are calling for public promotion of brain donation (Azizi et al. 2006; Eatough et al. 2012; Harris et al. 2013; Padoan et al. 2017). Understanding why people do or do not donate may assist professionals to coordinate and facilitate public education programmes about brain donation for research (Azizi et al. 2006).

We undertook a systematic review to identify what is known about factors influencing individuals’ or families’ decisions about brain donation for research purposes. Some research suggests that brain donation may hold more special personal significance for people than other organ donations (Boyes and Ward 2003; Nussbeck et al. 2015). To date, there has been no systematic assessment in this area.

This article reports on the results of a systematic review of the international literature that used qualitative and mixed methods research to identify people’s attitudes, motivations, and feelings about donating their or their loved ones’ brain to medical research. Our research question was: what are the factors people consider important in deciding whether or not to donate their or their loved ones’ brain for research? We limited the review to qualitative and mixed-methods research because we were interested in the voices of potential donors and their families which is strongly evident in these types of research.

## Methods

The review followed four procedural steps: (a) comprehensive search (b) quality appraisal, (c) synthesis of findings, and (d) critical appraisal.

### Search process

In May 2018 five electronic databases were searched: Scopus, PsycINFO, Embase, Medline, and Google Scholar. A systematic literature search method was utilised to capture the relevant studies that contained the desired terms in the title, abstract, or keywords. Search terms included: (“brain donor*” OR “brain donation” OR “brain banking” OR “banking on brain”) AND (attitude* OR motivation* OR decision*”) AND (LIMIT-TO “human”) AND (LIMIT-TO (LANGUAGE, “English”)).

### Inclusion and exclusion criteria

No limits were used for time or type of source. The exclusion terms were: (brain dead OR brain death).

Articles written in English were included. The final selection of articles was made using the following inclusion criteria: (1) the study explored the views/experience of potential brain donors about brain donation for research; (2) the study identified the views/experience of brain donors’ families about brain donation for research; (3) the study used qualitative or mixed methods approach.

**Fig. 1:**
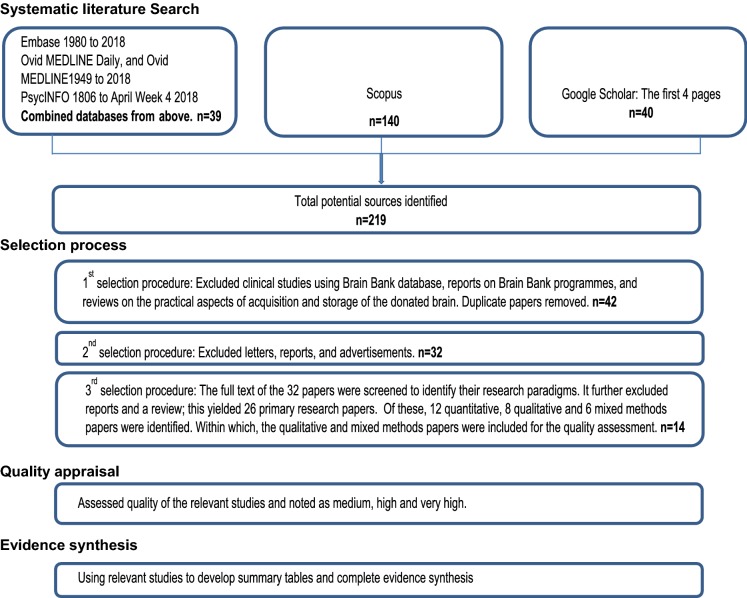
Flowchart of studies included in the literature review

The initial search identified 219 possible articles. Three rounds of the selection process (Fig. 1) were followed. First, we excluded clinical studies based on a Brain Bank database; reports on Brain Bank programmes; reviews on practical aspects of acquisition and storage of donated brains; and duplicate articles. Second, we excluded letters, reports and advertisements. Finally, the remaining articles were screened for relevance to our research questions.

### Evaluation of sources

#### Quality appraisal

We used the Framework for Assessing Qualitative Evaluation (FAQE) (Spencer et al. 2003) to appraise the quality of the included literature. The FAQE assesses methodological quality in terms of findings, design, sample, data collection, analysis, reporting, reflexivity and impartiality. We identified this tool as being especially useful for this review due to its appraisal questions and quality indicators for evaluation, which are specific to qualitative research. The FAQE has been used effectively for appraising qualitative research in other research (MacEachen et al. 2006).

#### Rigour

To ensure the suitability and credibility of FAQE, it was compared with the Standard for Reporting Qualitative Research checklist (SRQR) (O’Brien et al. 2014, p. 1245) where standards for reporting qualitative research are listed. We used both tools, FAQE and SRQR, on three of the articles selected for this review and compared findings. We decided to utilise the FAQE on the basis that it was more robust and detailed than the SRQR. This decision reflects MacEachen et al.’s (2006) assertion that FAQE acknowledges the “iterative and creative nature of qualitative research” because it offers a guideline for systematic consideration of papers rather than a checklist of the procedure (p. 258).

#### Qualitative focus

The FAQE focuses on qualitative research. We decided to use the FAQE and to focus on the review to qualitative and mixed-methods research because we were interested in the voices of potential donors and their families which is strongly evident in these types of research. Some of the excluded quantitative studies feature instead in the introduction and discussion sections of this paper.

#### Guiding principles

There are four central principles that underpin the content of the FAQE (Spencer et al. 2003). They advise that research should be:

1. Contributory in advancing wider knowledge or understanding about policy, practice, theory or a particular substantive field;
2. Defensible in design by providing a research strategy that can address the evaluative questions posed;
3. Rigorous in conduct through the systematic and transparent collection, analysis and interpretation of qualitative data; and
4. Credible in claim through offering well-founded and plausible arguments about the significance of the evidence generated. (p. 7)

These guiding principles include 18 appraisal questions (“Appendix 1”), each of which includes between four and seven quality indicators. The selected articles were reviewed independently by two members of the team (authors 1 and 2). These papers were assessed and noted to be medium, high, or very high in quality (see Table 1: Summary of study methods and foci for the validity assessment guidelines). The quality of the appraisal results was discussed by the two authors who conducted the appraisal to achieve concordance as a measure of the credibility of the data. If a consensus could not be reached, we planned for a third reviewer to be consulted. However, consensus was reached for each paper.

**Table 1:** Summary of the study methods and foci of the reviewed literature

Study	Location	Method	Participants	Recruitment	QA	Focus of study
1. Angelini et al. (2011)	Canada	Discussion Report of the institutional experience	27 Parents of children with Diffuse Intrinsic Pontine Glioma (DIPG) and these children in some cases	Children diagnosed with DIPG and treated at the Hospital of Sick Children	High	Donors’ families’ experience of post mortem brain or tumour donation and its impact on these families
2. Austrom et al. (2011)	USA	Community-based, participatory research model—focus group Semi-structured interview	30 Caregivers of persons with Frontotemporal dementia (FTD). The majority were spouses	Advertised via mailing of brochures and flyers to support group leaders and clinicians, and announcement on the Association for Frontotemporal Degeneration website and newsletter	High	To identify potential barriers to participate in brain donation programmes among FTD families To better understand the caregivers’ attitudes, awareness, and understanding of research and brain donation
3. Azizi et al. (2006)	Australia	Telephone interview	70 Next of kin. (48 were contacted. 22 could not be contacted.)	Through the Department of Forensic Medicine for the NSW Tissue Resource Centre or “brain bank”	High	To examine the verbal responses of the next of kin, on the day of autopsy, to the question of brain donation for medical research Specifically, to determine if next of kin find the question about brain donation on the day of autopsy distressing
4. Boise et al. (2017)	USA	Focus group semi-structured interview	61 African American, Chinese, Caucasian, and Latino individuals (potential brain donors and non-donors) and their 34 family members	Through focus groups at 4 NIH-funded Alzheimer’s Disease Centres	Very high	To explore beliefs and attitudes toward brain donation among 4 ethnic groups—African American, Chinese, Caucasian, and Latino research subjects and their families—from 4 funded Alzheimer’s Disease Centres
5. Eatough et al. (2012)	UK	Semi-structured interview Phenomenological informed thematic analysis	19 family members and friends of the brain donors. (14 spouses, 3 adult children, 1 sibling, 1 close friend)	Through a London brain bank	Very high	Examines beliefs, sense making and motivating factors concerning brain donation for research purposes
6. Garrick et al. (2009)	Australia	Mixed-methods Telephone call	Next-of-kin	Through the Department of Forensic Medicine	Medium	To explore factors that influence families’ decision to donate brain tissue to neuroscience research
7. Harris et al. (2013)	UK	Semi-structured one-on-one interview	19 potential brain donors (5 Parkinson’s disease, 14 unaffected)	Through PINE study	Very high	To identify factors people consider important when deciding whether or not to donate their brains for research
8. Jefferson et al. (2013)	USA	Mixed-methods Pre- and post-group survey	52 Older African Americans	Through the Boston University Alzheimer’s Disease Centre research registry	Very high	To implement an informational protocol for African Americans elders and their families about the benefits of clinical research and brain donation programme participation in AD. To assess participants’ changes in knowledge attitudes, and trust
9. Lambe et al. (2011)	USA	Focus group Consensual qualitative research method	15 African American older adults. (Their brain donation status include agree, do not agree, and undecided.)	Through Boston University Alzheimer’s Disease Core Centre research participation registry	Very high	To learn about African American older adults’ knowledge and perceptions of brain donation To learn about factors that related to participating or not participating in a brain donation programme To find ways to increase their brain donation commitment rates in the context of and Alzheimer’s disease research programme
10. Millar et al. (2007)	UK	Mixed methods	111 families of sudden death deceased	Medical Research Community Sudden Death Brain and Tissue Bank	Medium	To determine whether relatives who have been suddenly bereaved are willing to grant brain donation to a brain bank based in the forensic service
11. Padoan et al. (2017)	Brazil	In-depth semi-structured interview	18 participants: 12 patients with bipolar disorder and 6 family members.	A tertiary treatment program at the Hospital de Clínicas de Porto Alegre (the Bipolar Disorder Treatment Program)	Very high	To understand the attitudes and opinions of people treated for bipolar disorder and their relatives, regarding donation in general and donation for research
12. Schnieders et al. (2013)	USA	Structured face-to-face educational interview	91 African Americans aged 65 and older	Subsample of the Healthy Aging Research Study which was recruited from a database of registered voters	Medium	To recruit African American for a longitudinal aging study and to collect information about attitudes related to research To build trust and respect for research and to educate participants about the need for minority participants To identify barriers and incentives related to Alzheimer’s disease research and brain donation for African Americans
13. Stevens (1998)	UK	Mixed methods Letter–phone call or visit–questionnaire–interview	A subsample of 594 from a longitudinal Cognitive Function and Aging Study (CFAS) who were over 65 years old	People who were approached to consider brain donation and had made a decision about it Subsample of people over 65 selected randomly for the main CFAS in Nottingham in 1991	High	To find out the attitudes to brain donation for research purposes and factors involved in decision making in elderly people
14. Sundqvist et al. (2012)	Australia	Mixed methods Anonymous questionnaire-a follow-up questionnaire to the next-of-kin after the donation request was made on the day of their relatives’ coronial autopsy	111 Next-of-kin who consented to donate their relatives’ brains to the NSW Tissue Resource Centre (TRC) during the 6 year period from May 2002 to May 2008	Sourced from the Department of Forensic Medicine in Sydney	High	To further understand the families’ (who were contacted by telephone on the morning of their relatives’ coronial autopsy) motivation to donate brain tissue for medical research and to potentially help improve NSW TRC processes regarding the approach to next-of-kin to request brain donation Note: a follow-up research of previously described studies (Azizi et al. 2006; Garrick et al. 2009)

PINE study: an ongoing incidence and long-term follow-up study of Parkinson's disease in north-east Scotland

*QA* quality assessment

### Data extraction

As the data extraction progressed, the extracted information was stored in an Excel spreadsheet. The extraction included the focus of the research, method, theoretical orientation, sampling, context, analysis, findings, and researchers’ reflections. Extractions were initially conducted by the first author; later, meetings with the other authors were held to discuss the grouping of the themes. Data extracted from the selected sources were further arranged into two tables. Table 1: Summary of the study methods and foci; and Table 2: Factors influencing brain donation decision.

**Table 2:** Factors influencing brain donation decision of the reviewed literature

	Angelini et al. (2011)	Austrom et al. (2011)	Azizi et al. (2006)	Boise et al. (2017)	Eatough et al. (2012)	Garrick et al. (2009)	Harris et al. 2013)	Jefferson et al. (2013)	Lambe et al. (2011)	Millar et al. (2007)	Padoan et al. (2017)	Schnieders et al. (2013)	Stevens (1998)	Sundqvist et al. (2012)
*Motivations to donate (individuals and families)*														
Altruism—the desire to help others	**√**	**√**	**√**	**√**	**√**	**√**	**√**		**√**	**√**	**√**	**√**	**√**	**√**
Altruism—the desire to help medical research/‘gift of hope’	**√**	**√**	**√**	**√**	**√**		**√**			**√**	**√**		**√**	**√**
Altruism—the desire to help better understand the disease	**√**	**√**		**√**							**√**			
Altruism—personal or friends with a disease/prevent others suffering	**√**	**√**			**√**		**√**				**√**			
Altruism—family history of disease/to help future generation and others	**√**	**√**		**√**	**√**		**√**			**√**	**√**		**√**	
Altruism—a tiny step forward along with other people/to help others							**√**			**√**				
Gratitude for past treatment/positive health care experience		**√**			**√**				**√**	**√**			**√**	
Health literacy—knowledge about brain donation and research	**√**	**√**	**√**	**√**	**√**	**√**	**√**	**√**		**√**	**√**	**√**		
Health literacy—knowledge/experience participating in other research								**√**					**√**	**√**
Health literacy—exposure to medical, healthcare, and research settings	**√**			**√**				**√**	**√**		**√**	**√**		
Getting a definitive diagnosis		**√**			**√**		**√**		**√**					
Involved in group discussion about brain donation with family members		**√**						**√**						
Benefit to self or family members								**√**				**√**		
Fulfilment due to donation—death may become meaningful and helpful	**√**			**√**			**√**			**√**	**√**			
Education specifically about personal research benefits												**√**		
Communication—positive discussion with healthcare professionals	**√**	**√**			**√**							**√**		
Communication—early discussion about donation within the family	√	√		√	√					√		√		
It was the ‘right choice’ to help medical research/somebody has to do it			√		√								√	√
Religious beliefs					√		√							
Shan’t need brain/why destroy if it’s useful/recycling/avoid wastage					√								√	
Culturally sensitive approaches to brain donation				√				√	√					
Television or media													√	
*Motivation to donate (next*-*of*-*kin only)*														
Got some degree of comfort from making this donation	√		√							√				
Fulfilling, respecting or knowing deceased’s wishes	√		√		√	√								√
The opportunity to donate was empowering										√				
Wanted answers as to why the loved one had died										√				
*Barriers to donate (individuals and families)*														
Family against it/upset about it/consideration of other relatives’ wishes			√			√	√		√	√			√	
Emotional stress (discomfort about the idea of donation)	√			√		√							√	
Wish to keep the body whole			√	√		√							√	
Need brain/need to be whole in next life											√		√	
Can’t explain/no reason													√	
Can’t see the point—let others do it, I’ve done enough													√	
Lack of information—We want to help but don’t have enough information		√											√	
Lack of information about the brain donation procedure itself		√								√				
Fear—unknown (Don’t know enough about what happens after death)												√	√	
Fear—not being really dead/feeling pain after death													√	
Fear—integrity of the donor’s body/not knowing how the brain is used				√							√			
Fear—disfigurement	√			√										
Fear—knife cutting/too intrusive									√		√			
Fear—encountered with negative images							√							
Misconception—misunderstanding about brain donation procedures		√						√	√					
Misconception—not knowing normal brains needed				√					√					
Mistrust—racial discrimination in medical research (Tuskegee Study)				√				√	√					
Mistrust—racial disparities in medical settings (African American)				√				√	√			√		
Ineffective communication—negative experience with healthcare professionals		√			√		√		√	√				
Inappropriate timing and process when donation request was made	√	√			√					√				
Religious concerns	√	√	√	√		√					√	√	√	
Logistic issues—worry about the practicality of funeral arrangement	√	√		√		√								
Inconsistency among state law regarding powers of attorney (USA)		**√**												
*Barriers to donate (next*-*of*-*kin only)*														
Respecting or knowing the patient’s or deceased’s wishes/disliked	√	√	√			√				√			√	√
Conflict in the family about making a donation; therefore not donate			√											
Insufficient time to make a decision						√								
Retain image of deceased	√		√											
Next of kin was a young person who felt unprepared to decide										**√**				

### Synthesis of studies: meta-ethnographic approach

We used the meta-ethnographic approach to synthesise the data from articles included in this review. The meta-ethnographic analysis involves collating and synthesising findings from multiple published studies. The strength of this approach is that it applies a general interpretive lens for synthesising findings across multiple qualitative approaches (such as ethnography, grounded theory, or other interpretive approaches) and paradigms (Noblit and Hare 1988). It can also cater to qualitative data in diverse formats, such as interview transcripts, tabulated and descriptive notes, and matrices (Noblit and Hare 1988). It privileges comparison, interpretation, synthesis, and reciprocal translations of the meanings between and/or among the cases studied (Noblit and Hare 1988). This approach enables a rigorous procedure for interpretations about ethnographic and interpretive studies; it is “like the quantitative counterparts of meta-analysis” (Noblit and Hare 1988, p. 9). The meta-ethnographic approach has been used in other systematic reviews of the qualitative health literature (MacEachen et al. 2006). The meta-ethnographic approach “can lead to a synthesis and extension of qualitative research in a defined field of study” (Campbell et al. 2003, p. 671).

For the purpose of conducting a meta-ethnography, Noblit and Hare (1988) describe three different types of synthesis. The first is achieved through *reciprocal translation* when the concepts contained in the papers are similar and thus are directly comparable. The second is when the accounts stand in relative opposition, in which case a two-sided *refutational synthesis* can be engaged. In the third, the studies are combined to represent a *‘line of argument’ synthesis*, where repeated comparisons between studies were accomplished. In this review, data extraction provides the first form of synthesis. A two-sided *refutational synthesis* was used to discuss the contextual factors influencing decisions about the donation of the brain; they can be the motivations or barriers to donating. Later, following Glaser and Strauss (2017), our review reports on the findings from a synthesis of the selected studies, aiming to achieve a *‘line of argument’ synthesis* by recognising the similarities and differences among the studies.

## Findings

Fourteen articles were included for review, including nine qualitative (Angelini et al. 2011; Austrom et al. 2011; Azizi et al. 2006; Boise et al. 2017; Eatough et al. 2012; Harris et al. 2013; Lambe et al. 2011; Padoan et al. 2017; Schnieders et al. 2013) and five mixed methods studies (Garrick et al. 2009; Jefferson et al. 2013; Millar et al. 2007; Stevens 1998; Sundqvist et al. 2012). Of these, the FAQE identified six as very high quality (Boise et al. 2017; Eatough et al. 2012; Harris et al. 2013; Jefferson et al. 2013; Lambe et al. 2011; Padoan et al. 2017), five as high quality (Angelini et al. 2011; Austrom et al. 2011; Azizi et al. 2006; Stevens 1998; Sundqvist et al. 2012) and three as medium quality (Garrick et al. 2009; Millar et al. 2007; Schnieders et al. 2013) (Table 1). The earliest included article was published in 1998 by Stevens. Health literacy featured in all fourteen articles and altruism featured in thirteen articles as key motivations to donate (Table 2). Six articles described what motivated family (next-of-kin) to agree to the donation (Angelini et al. 2011; Azizi et al. 2006; Eatough et al. 2012; Garrick et al. 2009; Millar et al. 2007; Sundqvist et al. 2012). Thirteen articles identified deterrents to donation (excluding Sundqvist et al. 2012) and four of these stood out as listing considerably more deterrents and detail about them than other articles did (Austrom et al. 2011; Boise et al. 2017; Lambe et al. 2011; Stevens 1998). Commonly reported barriers to donation were ‘family against it’ (Azizi et al. 2006; Garrick et al. 2009; Harris et al. 2013; Lambe et al. 2011; Millar et al. 2007; Stevens 1998), ‘religious concerns’ (Angelini et al. 2011; Austrom et al. 2011; Azizi et al. 2006; Boise et al. 2017; Garrick et al. 2009; Padoan et al. 2017; Schnieders et al. 2013; Stevens 1998), and ‘ineffective communication with healthcare professionals’ (Austrom et al. 2011; Eatough et al. 2012; Harris et al. 2013; Lambe et al. 2011; Millar et al. 2007) (Table 2).

### Key concepts and meta-ethnographic synthesis

**Fig. 2:**
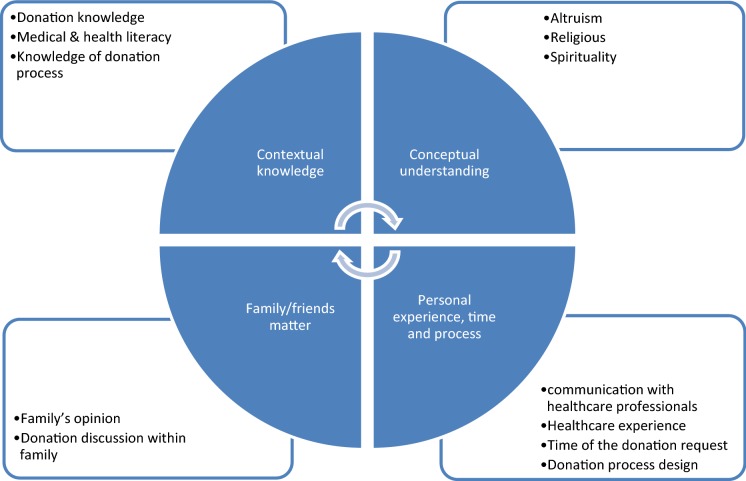
Our framework of how the brain donation decision works

Following the meta-ethnographic approach, our findings suggest four universal factors informing individuals’ or families’ decision to donate their brain: contextual knowledge, conceptual understandings, family/friends matter, and personal experience, time and process (Fig. 2).

### Contextual knowledge: health literacy

Bilbrey et al. (2018) assert that health literacy—the skills to access, read, process, understand, and communicate health related information—regarding brain donation can impact people’s decisions concerning brain donation. Although the health literacy of potential donors and their family members was not measured in any of the 14 included studies, participant knowledge about brain donation clearly varied from low to high. Participants who demonstrated high brain donation health literacy tended to describe their motivations to donate in terms of a desire to support research and to advance treatment (Angelini et al. 2011; Azizi et al. 2006; Boise et al. 2017). In the case of potential brain donors with healthy brain, Harris et al. (2013) explain that their participants understood “the valuable contribution the donated ‘normal’ tissue could make to the understanding of the pathological process” that underlie neurodegenerative disease (Harris et al. 2013, p. 1101). In contrast, five studies reported that prior to participation in their study participants had little knowledge about brain donation for research (Bilbrey et al. 2018; Boise et al. 2017; Harris et al. 2013; Jefferson et al. 2013; Stevens 1998). This finding is confirmed by broader literature: Garrick et al. (2006) write, “brain donation is a less familiar process to most of the population and is probably a more difficult personal decision that requires deliberation and consultation with loved ones” (p. 527).

Participants generally demonstrated more knowledge about organ donation for transplant purposes than brain donation for research purposes (Boise et al. 2017; Garrick et al. 2009; Harris et al. 2013; Stevens 1998). Some participants suggested that organ donation was (only) for anatomy education (Padoan et al. 2017). Others thought that the only use for donated organs was transplantation and this informed their ideas about whether donation was a viable path for them. For example, Stevens (1998) reports that many elderly respondents believed that no ‘parts’ of them would be ‘good’ for donation. Similarly, some people with bipolar disorder believed that they could not donate organs for transplantation because of their disease (Padoan et al. 2017). On learning about the prospect of brain donation, both sets of participants in these two studies were pleased to learn that they could be eligible to donate their brain for research purposes (Padoan et al. 2017; Stevens, 1998).

### Conceptual understandings

Donating one’s brain for research was referred to as a “gift of hope” by W. W. Tourtellotte, who initiated the collection of brain tissue for research in 1961 (Boyes and Ward 2003). All included articles presented potential donors’ and donors’ families’ views of organ donation as a categorically ‘good act’ and altruistic. The main reported motivation of participants across all 14 studies was desire to help others. A participant expressed the donation act as “a tiny step forward along with other people” (Harris et al. 2013, p. 1101) In other cases, families consented to donate based on the knowledge that autopsy is the only way to make a definite diagnosis of certain diseases and such knowledge—particularly of hereditary diseases—could be important for future generations (Austrom et al. 2011; Eatough et al. 2012). Boyes and Ward (2003) explain that most people with chronic illness and their carers “regard research as a source of hope for amelioration of the distress” caused by it, and that “becoming a brain donor gives them a sense of being able to contribute” to the community they depend on (p. 166). This finding is echoed by Azizi et al. (2006), who explain the principal motivation given by the participants was “because of the desire to help medical research” and that the donation gave a positive outcome to the death (p. 451).

In other cases, researchers assert that for parents of diffuse intrinsic pontine glioma (DIPG) children who consented to donation, the donation decision helped “to make sense of their child’s death, and fostered bereavement” (Angelini et al. 2011, p. 80). The families derived comfort from the hope that scientific breakthroughs could be made and felt that they were helping to make a difference in the advancement of the management of DIPG. In the case of two sets of parents, each set was particularly proud of their male child who expressed the wish to donate his brain to research. They shared memories with the team who treated their child and expressed appreciation to the palliative care team who offered end-of-life and home care. Angelini et al. (2011) conclude that the families’ meetings with the healthcare professionals, from the treatment to end-of-life care to the donation process, were important for bereavement purposes.

### Family/friends matter

The decision-making about brain donation for research takes place in two ways; either by the individual prior to death or by their family (usually, the next of kin) following death (Azizi et al. 2006; Boise et al. 2017). Family opinions—support or objections—for brain donation was well recognised across the 14 included studies as highly influential over donation outcome. Four included articles looked at this issue reasonably closely (Eatough et al. 2012; Harris et al. 2013; Lambe et al. 2011; Stevens 1998). Two studies questioned whether the individual or family’s preference should prevail (Boise et al. 2017; Harris et al. 2013). One study found that some donors resented the possibility that their children could or might influence the donation outcome (Stevens 1998).

Multiple studies indicated that family discussion and making the decision prior to the individual’s death promoted positive donation outcome and positive family experience (Azizi et al. 2006; Boise et al. 2017; Harris et al. 2013; Lambe et al. 2011; Stevens 1998). Azizi et al. (2006) state that “knowledge of the deceased’s wishes regarding organ donation was the main reason given by families when making their decision” to give or deny consent for brain donation (p. 451). In one study, a donor’s husband, Peter recalled:

For one short moment … I had a sudden thought should I go ahead with it or shouldn’t I … I’m glad that I didn’t waver and in fact I went ahead with her wishes and that I think it was the right thing for me (Eatough et al., 2012, p. 1280).

This example is consistent with the finding that having enough information about the deceased’s wishes increased family satisfaction with the decision (Stevens 1998). Sundqvist et al. (2012) write, “Almost a quarter (24%) commented that they had decided to donate because they were either aware that their deceased relative had wanted to be an organ donor, or believed it was something he or she would have wanted” (p. 95). Discussions within the family and knowing the deceased’s wishes were also reported as influential in coronial autopsy settings where the donation of brain tissue to medical research was requested on the day of autopsy (Azizi et al. 2006; Sundqvist et al. 2012).

In some instances, the brain donation decision was made based on “a shared implicit understanding”, values, and beliefs between the individuals and their family members (Eatough et al. 2012, p. 1279).

### Personal experience, time and process

Personal experience, including healthcare and hospital experience, and the manner of the donation invitation/request, affects people’s donation decision (Angelini et al. 2011; Austrom et al. 2011; Azizi et al. 2006; Eatough et al. 2012; Sundqvist et al. 2012). The combination of the quality and timeliness of the invitation/request, and the environment in which the invitation is given/received influenced the response. For example, Eatough and colleagues report the experience of a non-donor participant who received a donation request via her mobile phone while in a supermarket car park. Despite the participant suggesting a call back, the clinician insisted on carrying on. The participant reflected on the conditions that might have encouraged people to donate; “you’d have to be with somebody and see their face, look at them, and get to know them a little bit even … There’s certain things you can’t say to people without prejudging a little bit beforehand and sizing them up to find out if they can take it” (Eatough et al. 2012, p. 1278).

In other cases, families felt that the funeral directors did not take the donation act seriously. Elizabeth, wife of a donor, recalled:

I said his brain has to go for research, now, I actually got the forms out and this bloke looked at me as much as to say you’ll be lucky … I’m really upset about this because the only wish Bill had got in his life was that his brain was to go for research. I had a fight on my hands … I had to do as he wished, I pushed heaven and earth (Eatough et al., 2012, p. 1281).

Based on such accounts, Eatough and colleagues urged that quality of communication and practice (including processes) amongst relevant healthcare professionals can be critical to informing donation outcome. They state that “Healthcare and related professionals need to be aware of the significance of [the donation] act and recognise their responsibility in ensuring that the process brings comfort rather than distress”, and argue for “the need for privacy and empathy” when the donation invitation is made (Eatough et al. 2012, p. 1278).

In contrast, a positive experience of the brain donation request can encourage consent to donate. For example, a potential donor describing the healthcare professional who invited them exclaimed, “I couldn’t have wished for anyone kinder” (Eatough et al. 2012, p. 1278). In another study, a family member expressed appreciation that the researchers gave the family information about brain donation and research ahead of time: “… had she just passed, and then you guys brought a paper to me saying, ‘Your mum’s agreed to donate,’ that would be very hard for me… So I appreciate you guys taking the time to do this” (Boise et al. 2017, p. 724). Thus, the importance of providing potential donors and their families a positive communication experience—in a timely manner—is emphasised (Azizi et al. 2006; Eatough et al. 2012).

One included study reported that several next of kin participants said they would have liked more time to make decisions (Sundqvist et al. 2012). Timing for families and for researchers is important. The researchers explain that those next of kin contacted by the NSW Tissue Resource Centre in Australia during their relative’s autopsy are almost always required to make the donation decision within a 2 h period from 9.00 AM; “a time frame dictated by the post-mortem examination itself” (Sundqvist et al. 2012, p. 98).

Potential donors and their families reported feeling anxious about the logistics of brain donation and funeral arrangements (Austrom et al. 2011; Boise et al. 2017; Eatough et al. 2012; Harris et al. 2013). Some study participants were worried that following the brain donation their loved one’s body would appear disfigured and this would be noticeable at an open-coffin funeral (Angelini et al. 2011; Boise et al. 2017; Garrick et al. 2006). Others experienced delays with the doctor issuing a death certificate, and healthcare professionals being insensitive and/or lacking awareness about their situation (Eatough et al. 2012). One study participant—a donor’s friend called Cynthia—experienced procedural delays and recounted feeling angry with the nursing staff. Cynthia recounted, “I’ve got to do this for her. I was very aware that this was something, the job she’d asked me to do and I had to do it and these people saying well, we’ve got to wait you know you’ve got to wait there isn’t a doctor on duty” (Eatough et al. 2012, p. 1281).

## Discussion

This review set out to identify factors informing people’s decisions to donate their own or their loved one’s brain for research. We identified 14 articles that contribute to answering this question. Overall, the quality of the included literature was very high or high and this is encouraging as it lends weight to the meta-ethnographic findings. Four universal factors were identified in the literature that inform a person’s brain donation decision: contextual knowledge, conceptual understandings, family/friends matter, and personal experience, time and process (Fig. 2). These factors do not stand alone. Rather, they interconnect to inform the complex psychological and social processes behind a person’s decision.

We found people’s contextual knowledge (which we discuss in terms of health literacy) informs people’s donation decisions. Studies often discussed contextual knowledge in terms of participant ‘understanding and misunderstanding(s).’ Some studies reported on how participant knowledge, values and ideas informed donation outcome rather than on how participant beliefs informed donation outcome (although six articles did list religious beliefs as a barrier to donation). In terms of values, for example, Eatough and colleagues explain that for their participants, “the brain is not special but its presence on a laboratory shelf has *meaning.*” (Eatough et al. 2012, p. 1283). The *meaning* that participants attributed to brain donation for research served to facilitate their grieving processes and offered them a degree of comfort.

Our second finding relates to conceptual understanding of brain donation as a ‘good’ or altruistic act. Gawande writes, “the only way death is not meaningless is to see yourself as part of something greater: a family, a community, a society. If you don’t, mortality is only a horror. But if you do, it is not” (Gawande 2014, p. 127). This sentiment resonates with how donors and their families conceptualised brain donation in our reviewed literature. Potential donors expressed altruistic motivations in their decision-making processes and in conversations with family members. People saw brain donation as a way to help others, their family members and descendants, other people with the same condition, or society in general. Brain donation was also a source of comfort and hope for family members. Healthcare professionals strongly influenced people’s experiences of donation processes.

Our third finding highlights the influence of family and importance of their involvement in the donation decision process. Knowledge of a family member’s wishes prior to their death influenced family members’ decisions and donation outcomes. Conversations about donation between potential donors and their family or friends were therefore identified as very important. Such conversations may relieve potential donors’ anxiety that their family will not honour their donation decision. Increasing contextual knowledge and health literacy of family members may positively inform donation outcomes.

Our fourth and final key finding is that personal experience, time and process are the external factors that affect donation decisions. The conditions under which the invitation to donate is made influence a potential donors’ decision. The literature indicates that a well-timed and personal invitation, received in an appropriate environment is important. In the context of the coronial autopsy, Garrick et al. (2009) found that the timing of the brain donation request influenced their consent rates. In their study, most donation requests were made within 60 h of the potential donor’s death. Their study found that the longer the interval between death and the donation request, the more likely a consent was gained. They explained that families need time to come to terms with the death of their relatives before being approached about donation of organs or tissue (Garrick et al. 2009), which is consistent with other literature (West and Burr 2002). Trujillo Diaz et al. (2018) have recently demystified the processes that support such conversations and donation outcomes. Their article additionally provides a wealth of guidance for effective management of the various processes involved—from advertising, screening and consenting to donor communication and brain harvest processes.

In instances where a donation decision needs to be made quickly (following death), it is important to provide the potential donor’s family with information—in the right amount and pitched at the right health literacy level—about donation for research. This provision may be highly influential over donation outcome. For a successful donation outcome these factors must all align: the donor’s family has a shared understanding of the donor’s wishes, are well informed and do give permission; donation processes are followed in a smooth and timely fashion, and donation must proceed without undue stress toward the donor or their family. This is particularly pertinent given that the donation processes largely occur while family members are in a raw and intense stage of grief over the death of their loved one. We, therefore, urge those involved in brain donation processes to be mindful and empathetic of people’s feelings in each of these areas.

What this review is largely missing are the voices of people with healthy brain who are considering brain donation. To what extent are their motivations the same? Population survey research concerning people’s brain donation health literacy could help us answer our questions about healthy brain donation motivations and barriers, as could qualitative research with people who have healthy brain about their views, values, and beliefs that inform their donation decisions.

Many factors influence people’s donation decision. We have detailed the factors described in the literature. However, gaps remain in what we know. How do people sustain motivation for donation? Although some people maintain their motivation for years and in the context of complex donating processes, we know little about how they do this. We also know little about the factors informing the donation of young and/or healthy brains. In this review, only one article focused on the donation of brains from young children. Some different issues arise in that context than from adult brain donation. For example, the relationships between the donors and their family are different, and the agency is differently located. We call for more research that can fill this age-related research gap. In this paper, we have presented findings on voluntary donation and donation through coronial autopsy (see Table 2). Again, different processes and issues are involved, and we know little of how such differences inform donation decisions. Qualitative research in this area would be valuable to those working in brain donation-related fields.

### Meta-ethnographic synthesis process

Meta-ethnographic synthesis approach followed for this review enables a rigorous procedure for interpretations about ethnographic and interpretive studies (Noblit and Hare^1988^). In this systematic review, the research question about the factors people consider important in deciding whether or not to donate their or their loved ones’ brain for research guided our inquiry of the paper reviewed. We found that the compilation of findings in key concepts (Table 2) provided a detailed mapping of the influencing factors. These key concepts then provided a platform for analysis and synthesis that extends beyond what an empirical study can offer. Based on this mapping we developed our conceptual framework (Fig. 2) to better understand how the donation decision processes work.

## Limitations

This research only included studies written in English. Studies that only included quantitative research methods were excluded due to our analytical methods. This meant that some important literature was not included in the findings but we have referred to them in our introduction and discussion.

The search term ‘brain donation’ was used in place of ‘organ donation’ because we specifically wanted to explore brain donation rather than any other organ donation. It is possible that articles may have been missed where they did not mention brain donation in the title, keywords, or abstract.

## Conclusions

This review included fourteen articles, six of which were appraised as very high quality. We asked, what are the factors people consider important in deciding whether or not to donate their or their loved ones’ brain for research? The factors are the contextual knowledge (brain donation health literacy) and conceptual understandings of potential donors and their families (such as altruism); family influences and needs; personal experiences (including conversations with healthcare and donation professionals); time (timing of conversation and of process completions); and donation and funeral processes. These many and complex factors need to align for a positive donation outcome.

## Appendix 1: Key questions in the Framework for Assessing Qualitative Evaluations (FAQE)

### Findings

1. How credible are the findings?
2. How has knowledge/understanding been extended by the research?
3. How well does the evaluation address its original aims and purpose?
4. Scope for drawing wider inference—how well is this explained?
5. How clear is the basis of evaluative appraisal?

### Design

6. How defensible is the research design?

### Sample

7. How well defended is the sample design/target selection of cases/documents?
8. Sample composition/case inclusion—how well is the eventual coverage described?

### Data collection

9. How well was the data collection carried out?

### Analysis

10. How well has the approach to, and formulation of, the analysis been conveyed?
11. Contexts of data sources—how well are they retained and portrayed?
12. How well has diversity of perspective and content been explored?
13. How well has detail, depth, and complexity (i.e. richness) of the data been conveyed?

### Reporting

14. How clear are the links between data, interpretation, and conclusions—i.e. how well can the route to any conclusions be seen?
15. How clear and coherent is the reporting?

### Reflexivity and neutrality

16. How clear are the assumptions/theoretical perspectives/values that have shaped the form and output of the evaluation?

### Ethics

17. What evidence is there of attention to ethical issues?

### Auditability

18. How adequately has the research process been documented?
